# Laparoscopic management of mesh migration into urinary bladder following laparoscopic totally extraperitoneal inguinal hernia repair—A case report

**DOI:** 10.1016/j.ijscr.2020.12.065

**Published:** 2020-12-28

**Authors:** Kishor R J, Kuppan C T, Narayanan Cunnigaiper Dhanasekaran, Vishnu Sekar

**Affiliations:** Department of General Surgery, Sri Ramachandra Institute of Higher Education, Porur, Chennai, Tamil Nadu, 600116, India

**Keywords:** TEP, Totally Extra Peritoneal repair, TAPP, Trans Abdominal Pre-Peritoneal repair, UTI, Urinary Tract Infection, KUB, Kidney, Ureter, Bladder, POD, post-operative day, Inguinal hernia repair, Mesh migration, Mesh erosion, Urinary bladder, Complication, Laparoscopy

## Abstract

**Introduction and importance:**

Mesh migration into urinary bladder is one of the rare complications following inguinal hernia repair (Laparoscopic/Open). On reviewing the literature, erosion of mesh following inguinal hernia repair has been into the urinary bladder in most of the cases, and the erosion may occur as early or late complication. It may occur as a result of improper suturing, inadequate fixation or foreign body reaction. The most common presentation is recurrent urinary tract infection and haematuria and may mimic bladder malignancy.

**Case presentation:**

A 38-year male presented with recurrent UTI and mimicked to have bladder malignancy on CT scan. On Cystoscopy, mesh along with tackers is visualized within the bladder lumen. A diagnosis of Mesh migration into bladder following laparoscopic inguinal hernia repair was made. The Patient underwent Complete laparoscopic removal of mesh with partial cystectomy, per urethral and suprapubic catheter were placed. The patient made a good recovery without any post-operative complications. On follow-up, Patient underwent Fluoroscopy to look for urinary leakage, and suprapubic catheter removal was done. Patient is asymptomatic on follow-up.

**Clinical discussion:**

Mesh migration into bladder is one the rare complications following laparoscopic hernia repair. Proper preoperative evaluation is necessary to determine whether mesh is free floating in the bladder lumen or adherent to bladder wall. This will help in deciding the surgical technique for route of extraction.

**Conclusion:**

A case of mesh migration into the bladder can be easily managed by laparoscopic TAPP approach and it is better approach compared to other techniques.

## Highlights

• Mesh migration into bladder should be considered a differential in case of patients with recurrent UTI and history of laparoscopic hernioplasty.
• Computed tomography and cystoscopy will aid in the diagnosis of such cases and removal by TAPP approach is better than other approaches.
• This case report will help the readers to get a knowledge on such an approach as it is easily reproducible.
• Care should be taken to remove all the mesh as remnant mesh particles may act as a nidus for infection.

## 1 Introduction

Surgical repair is the gold standard treatment of hernias regardless of their location. With advancement in minimal access surgery, laparoscopic hernia repair became an alternative approach to open surgery in reducing post-operative complications. Incidence of complications following laparoscopic inguinal hernia repair is rare, Trocar site bleed and bowel injury are significant complications occurring in 0.4%–5.6%. Mesh infection occurs in 0.1% [1]. Migration of mesh into visceral organs following laparoscopic inguinal hernia is one of the rare complications occurring following laparoscopic hernia repair. We report a case of mesh migration into urinary bladder following Totally Extra Peritoneal repair, which was managed laparoscopically in accordance with SCARE 2020 criteria [2].

## 2 Case report

**Fig. 1:**
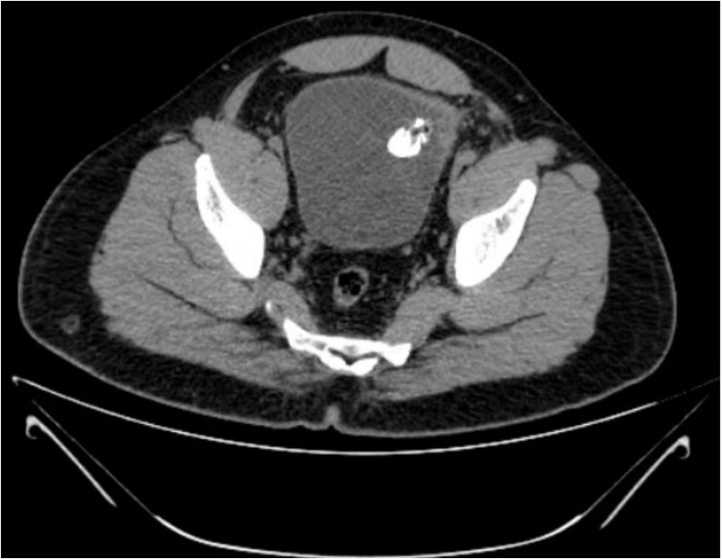
CT scan showing intraluminal calcified polypoid lesion.

**Fig. 2:**
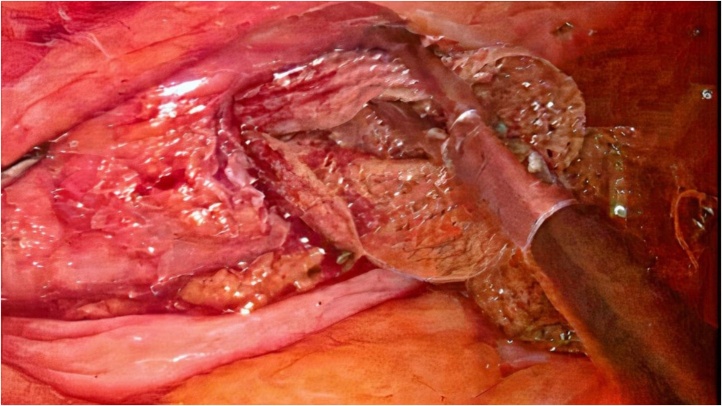
Intraoperative image showing mesh adherent to bladder wall.

A 38-year-old male with no known comorbidities and a history of laparoscopic inguinal hernia repair (Totally Extraperitoneal) presented with complaints of recurrent UTI infection. On Clinical examination, expansile cough impulse was present in the left inguinal region. Urinary culture showed Pseudomonas species growth and was treated with culture-sensitive antibiotics. The Patient was evaluated further due to persistent symptoms of Urinary tract Infection. Ultrasound scan of Kidney, Ureter and Bladder (KUB) showed multiple calculi in the urinary bladder, obstructing vesicoureteric junction. Computed tomography done suggested intraluminal calcified polypoidal lesion with focal wall thickening with surrounding fat stranding in the anterolateral aspect of the urinary bladder wall (Fig. 1). On Cystoscopy mesh was identified within the bladder lumen with metal tackers. The Patient planned for Laparoscopic mesh removal, intraoperatively mesh found adherent to the bladder wall (Fig. 2) and was proceeded with partial cystectomy with mesh removal and repair of the bladder in two layers was done. A suprapubic catheterisation fixed through the laparoscopic port in addition to the urethral catheter, and a drain placed. Drain removal done on POD 10. On POD 16 fluoroscopy done showed no leakage and the suprapubic catheter removal done. Patient was asymptomatic on follow-up and made an unremarkable recovery. On examination wound found to have healed wounds without any complications.

## 3 Discussion

Laparoscopy has gained worldwide acceptance in the management of inguinal hernia. In a Cochrane study involving 41 trials with 7161 participants, the authors concluded that while laparoscopy offered significant benefits of early return to work, less persisting pain and numbness, there is a significant rise in visceral complications of serious nature especially bladder [3].

In an overview of published reports on Mesh erosion into the urinary bladder, a total of 21 cases of mesh erosion were recorded of which 11 cases were following laparoscopy (TEP or TAPP), and in four cases the laparoscopic procedure was not mentioned. There was only one documented case following Liechtenstein repair [4].

The reason for the mesh eroding into the viscera might be its close proximity to the colon or bladder. In laparoscopy - either TEP or TAPP approach the mesh is placed in the pre-peritoneal space, whereas Liechtenstein repair a commonly performed open technique involves onlay placement of the mesh for inguinal hernia.

Laparoscopy has a steep learning curve, and faulty techniques are likely to play a role in mesh migration. The placement of mesh is tailor-made in open inguinal hernia repair which helps in securing the mesh with no crumbling, but in laparoscopy, the dissected space may be out of proportion with the size of the mesh leading to folding and crumpling of the mesh which may predispose to mesh migration. Although non-fixation of mesh has an advantage of less neuralgic pain, it may cause mesh migration and erosion into adjacent visceral organs.

The mechanism leading to mesh migration can be subdivided into primary (early) due to mechanical factors or secondary (late) due to inflammatory factors [5].

The primary mesh migration is mainly due to mechanical factors such as folding or crumpling of the mesh that results due to inadequate dissection, improper fixation or no fixation of mesh, type of the material used, sharp edges of metal tackers, the weight of the mesh (lightweight can contribute to primary migration whereas heavyweight can contribute to secondary migration) and most importantly faulty technique. The migrated mesh does not get incorporated into the surrounding tissues; hence removal of these are technically easier.

The secondary mesh migration happens over a period of months to years gradually and can be described as a bubble effect - where the bubble gradually moves, enclosing within it the mesh, towards the path of least resistance which is either the lumen of a viscera or the skin. In a review of literature by Li and Cheng [4] the time interval between the hernia repairs to the presentation of mesh erosion into bladder varied between one year to twenty years. In our case, it took ten years to manifest clinically. There is a zone of intense inflammation in the lead point, which is thought to be a foreign body reaction. The prolonged duration between implantation and explantation suggests other possibilities of immune-mediated or simmering chronic infections. In our Patient the swab taken from the mesh showed pseudomonas growth could have been due to exposure of mesh in the urinary bladder lumen. If the mesh completely erodes into the viscera, it can be retrieved by endoscopic means [5]. In case of partial erosion of mesh, the patient can present with varied clinical presentations depending on the organ and extent of erosion. In our Patient the clinical picture of hematuria and the polypoidal mass made us suspect a malignancy and the tackers mimicked calcification in a neoplasm very similar to one described by Sevilla [6].

The migrated mesh can get totally integrated into the tissue it reaches, resulting in resection of segment of the viscera it migrates [7]. Usually, the peritoneum forms a natural barrier preventing adhesions between the bowel and the mesh. If there is a breach or raw area on the serosa of the bowel, adhesions form and later aided by peristalsis cause the mesh to migrate. Sometimes it shrinks and forms a ball aptly named meshoma. Several treatment options have been described in the literature majority of which is open surgery with partial or complete removal of the mesh [8,9] or via Transurethral approach [10].

Laparoscopic TEP approach [11] was used recently to retrieve a portion of the mesh, and the author had quoted three advantages of TEP plane over TAPP plane. First being virgin route to approach the mesh erosion site. However, we do not agree because whether it is TEP or TAPP only the access route is different in both techniques, the mesh is placed in the preperitoneal space only. Secondly only partial removal of mesh was done leaving behind the unmigrated portion which we feel is not the correct intent as leaving behind mesh which is not integrated will lead to further sinus formation, erosion and a nidus for Infection. The third reason given was the exposure. We preferred the TAPP approach as it gives an excellent view both of the bladder wall and pelvis. It was also easy to do a partial cystectomy where the mesh had become integrated with the bladder wall, and intracorporeal suturing is technically easier with TAPP as it allows good triangulation. Hence, this case provides a key learning opportunity for all the readers as it helps the clinicians in choosing the TAPP approach over other approaches in managing such cases. Despite the increasing incidence of mesh migration into bladder following inguinal hernia repair, surprisingly, no cases have been reported of TAPP approach for management in the literature.

## 4 Conclusion

This case highlights TAPP approach as it is safe, technically feasible and reproducible, allowing early ambulation. Secondly, a high index of suspicion should be entertained in patients who complain of urinary symptoms following laparoscopic inguinal hernia; however distant they may seem. Thirdly maintenance of registry by hernia societies for mesh-related events globally is necessary as it would then give a clear picture of the risk-benefit ratio.

## Declaration of Competing Interest

The authors report no declarations of interest.

## Sources of funding

Nil.

## Ethical approval

Not required.

## Consent

Written informed consent was obtained from the patient for publication of this case report and accompanying images. A copy of the written consent is available for review by the Editor-in-Chief of this journal on request.

## Author contribution

Kishor R J – Data collection, Writing the manuscript, Study design.

Kuppan C T – Data collection, Writing the manuscript, Study design.

Narayanan C D – Data collection, Writing the manuscript, Study design, Final draft correction and review.

Vishnu Sekar – Involved in the case.

## Registration of research studies

Not applicable.

## Guarantor

Narayanan C D.

Kishor R J.

## Provenance and peer review

Not commissioned, externally peer-reviewed.
